# Predictors for Survival in an International Cohort of Patients Undergoing Distal Pancreatectomy for Pancreatic Ductal Adenocarcinoma

**DOI:** 10.1245/s10434-020-08658-5

**Published:** 2020-06-25

**Authors:** M. Korrel, S. Lof, J. van Hilst, A. Alseidi, U. Boggi, O. R. Busch, S. van Dieren, B. Edwin, D. Fuks, T. Hackert, T. Keck, I. Khatkov, G. Malleo, I. Poves, M. A. Sahakyan, C. Bassi, M. Abu Hilal, M. G. Besselink, for the European Consortium on Minimally Invasive Pancreatic Surgery (E-MIPS)

**Affiliations:** 1 Department of Surgery, Cancer Center Amsterdam, Amsterdam UMC, University of Amsterdam, Amsterdam, The Netherlands; 2 Department of General Surgery, Instituto Ospedaliero Fondazione Poliambulanza, Brescia, Italy; 3 Department of Surgery, OLVG Oost, Amsterdam, The Netherlands; 4 Division of Hepatopancreatobiliary and Endocrine Surgery, Virginia Mason Medical Center, Seattle, WA USA; 5 Division of General and Transplant Surgery, University of Pisa, Pisa, Italy; 6 Department of HPB Surgery, The Intervention Center, Institute for Clinical Medicine, Oslo University Hospital, Oslo, Norway; 7 Department of Surgery, Institut Mutualiste Montsouris, Paris, France; 8 Department of Surgery, Heidelberg University Hospital, Heidelberg, Germany; 9 Department of Surgery, University Hospital Schleswig–Holstein, Campus Lübeck, Lübeck, Germany; 10 Department of Surgery, Moscow Clinical Scientific Center, Moscow, Russian Federation; 11 Department of Surgery, Pancreas Institute, Verona University Hospital, Verona, Italy; 12 Department of Surgery, Hospital del Mar, Barcelona, Spain; 13 Department of Surgery N1, Yerevan State Medical University After M. Heratsi, Yerevan, Armenia

## Abstract

**Background:**

Surgical factors, including resection of Gerota’s fascia, R0-resection, and lymph node yield, may be associated with survival after distal pancreatectomy (DP) for pancreatic ductal adenocarcinoma (PDAC), but evidence from large multicenter studies is lacking. This study aimed to identify predictors for overall survival after DP for PDAC, especially those related to surgical technique.

**Patients and Methods:**

Data from an international retrospective cohort including patients from 11 European countries and the USA who underwent DP for PDAC (2007–2015) were analyzed. Cox proportional hazard analyses were performed and included Gerota’s fascia resection, R0 resection, lymph node ratio, extended resection, and a minimally invasive approach.

**Results:**

Overall, 1200 patients from 34 centers with median follow-up of 15 months [interquartile range (IQR) 5–31 months] and median survival period of 30 months [95% confidence interval (CI), 27–33 months] were included. Gerota’s fascia resection [hazard ratio (HR) 0.74; *p* = 0.019], R0 resection (HR 0.70; *p* = 0.006), and decreased lymph node ratio (HR 0.28; *p* < 0.001) were associated with improved overall survival, whereas extended resection (HR 1.75; *p* < 0.001) was associated with worse overall survival. A minimally invasive approach did not improve survival as compared with an open approach (HR 1.14; *p* = 0.350). Adjuvant chemotherapy (HR 0.67; *p* = 0.003) was also associated with improved overall survival.

**Conclusions:**

This international cohort identified Gerota’s fascia resection, R0 resection, and decreased lymph node ratio as factors associated with improved overall survival during DP for PDAC. Surgeons should strive for R0 resection and adequate lymphadenectomy and could also consider Gerota’s fascia resection in their routine surgical approach.

**Electronic supplementary material:**

The online version of this article (10.1245/s10434-020-08658-5) contains supplementary material, which is available to authorized users.

Pancreatic ductal adenocarcinoma (PDAC) is associated with a very poor 5-year survival of 5–10%.^1^,^2^ Surgical resection combined with adjuvant chemotherapy provides overall survival periods up to 24–32 months.^3^^–^5 The identification of surgical, histopathological, and oncological predictors for overall survival is important for guidance in treatment options and optimal prognostication for patients affected by PDAC.

In the last decade, multiple studies have suggested predictors for overall survival in patients undergoing distal pancreatectomy (DP) for PDAC. These predictors included margin status, lymph node dissection, and adjuvant chemotherapy as positive predictors; and tumor stage, tumor grade, tumor size, lymphovascular and perineural invasion, lymph node metastases, and increasing lymph node ratio as negative predictors.^3^,^4^,^6^^–^10 The majority of the identified predictors for overall survival are tumor related and cannot be influenced by surgeons. Very few of these studies addressed variables related to surgical technique such as Gerota’s fascia resection, minimally invasive surgery, and multivisceral resection. However, these variables may be associated with improved survival, but data are lacking.^11^,^12^ The relevance of surgical technique for survival after distal pancreatectomy for PDAC has not yet been studied in multicenter international studies.

The primary objective of this study is to identify predictors for overall survival related to surgical technique, including Gerota’s fascia resection, R0 resection margin, lymph node ratio, extended resection, and a minimally invasive approach in a large international multicenter cohort of patients undergoing distal pancreatectomy for PDAC.

## Patients and Methods

This study was performed according to the Strengthening the Reporting of Observational studies in Epidemiology (STROBE) and the Transparent Reporting of a multivariable prediction model for Individual Prognosis or Diagnosis (TRIPOD) guidelines for Prediction Model Development.^13^,^14^

### Patients and Design

This is a post hoc analysis of the previously published international multicenter retrospective DIPLOMA cohort, which involved high-volume pancreatic centers of the European Consortium on Minimally Invasive Pancreatic Surgery (E-MIPS).^15^ All consecutive patients who underwent either minimally invasive or open distal pancreatectomy for PDAC between January 1, 2007 and July 1, 2015 were included. Patients who died within 90 days after surgery and those with metastasized disease at the time of surgery were excluded.

### Data Collection and Definitions

The variables included in the analyses were defined as presented in the published DIPLOMA cohort.^15^ A list of all variables can be found in the Appendix. Minimally invasive distal pancreatectomy was defined as laparoscopic or robot-assisted distal pancreatectomy. Extended distal pancreatectomy was defined according to the International Study Group for Pancreatic Surgery (ISGPS) definition and included multivisceral resections beyond the pancreas and spleen and/or vascular resections beyond the splenic vessels.^16^ For tumor stage, the seventh classification of the American Joint Committee on Cancer (AJCC) was used.^17^ Resection margins were assessed at both the circumferential and transection margins. Circumferential margins were defined as either the posterior margin or the anterior, superior, and inferior surfaces of the pancreas. Resection margins were divided into three categories: R0 (tumor ≥ 1 mm from margin), R1 (tumor < 1 mm from margin), and R2 (macroscopically positive margin) according to the Royal College of Pathologists definition.^18^ During data collection, the exact distance in millimeters from tumor to margin was collected, which was then classified as either R0, R1, or R2 by the study coordinator. Major complications were defined as Clavien–Dindo grade III or higher.^19^ Data on Gerota’s fascia resection were collected from surgical notes. Centers routinely resecting Gerota’s fascia were (arbitrarily) defined when this was done in > 90% of all included patients. Administration of adjuvant chemotherapy was defined as starting any type of adjuvant chemotherapy regimen.

### Primary and Secondary Outcomes

The primary endpoint of this study was median overall survival. Survival data were collected for all patients until their latest oncologic surveillance examination or until death, based on patient files and the municipal records database. The secondary endpoints included the assessment of aforementioned intraoperative, postoperative, histopathology, and oncology outcomes.

### Statistical Analyses

Data were analyzed using IBM SPSS Statistics for Windows version 26.0 (IBM Corp., Orchard Road Armonk, New York, NY). Categorical data are presented as percentages and frequencies. Normally distributed continuous data are presented as means and standard deviations (SDs). Non-normally distributed continuous data are presented as medians and interquartile ranges (IQRs). Dichotomous data were compared using a *χ*^2^ analysis, while continuous data were compared using an independent Student’s *t* test or Mann–Whitney *U* test as appropriate.

Survival was assessed using a Kaplan–Meier analysis from the date of distal pancreatectomy until death or last moment of follow-up (censored observation). Survival among subgroups (e.g., patients who underwent minimally invasive distal pancreatectomy versus patients who underwent open distal pancreatectomy) was assessed by stratified Kaplan–Meier analyses. Univariable and multivariable Cox proportional hazard analyses with backward selection were performed to identify surgical predictors for survival. Variables with *p* value < 0.1 on univariable analysis were all included in one single multivariable Cox proportional hazard model^20^ with forced entry of surgical approach (minimally invasive or open distal pancreatectomy). In this model, other well-known predictors for survival were incorporated, thus adjusting the analysis for their effect on survival. For all variables selected in the univariable and multivariable models, the proportional hazards assumption was tested by constructing log minus log plots. Results of the Cox proportional hazard analyses are presented in hazard ratios (HR). All confidence intervals (CI) are presented at the 95% significance level.

Sensitivity analysis was performed by excluding the 25% largest tumors, since Gerota’s fascia resection could be less effective when resecting smaller tumors. Subgroup analysis comparing patients who received Gerota’s fascia resection with patients who did not receive Gerota’s fascia resection was also performed. Two-tailed *p* value lower than 0.05 was considered statistically significant.

## Results

### Cohort Characteristics

Data were collected for 1296 patients who underwent distal pancreatectomy for PDAC in 34 centers from 12 countries (11 in Europe plus the USA). Overall, 34 (2.6%) patients who died within 90 days postoperatively, and 62 (4.8%) patients with metastatic disease at time of surgery were excluded, leaving 1200 patients with median age of 68 (IQR 61–75) years for further analyses. Neoadjuvant treatment was administered in 10.7% (*n* = 128) of patients.

### Surgical and Postoperative Outcomes

Of all procedures, 70.7% (*n* = 848) were performed by an open technique. The majority of the minimally invasive distal pancreatectomy procedures were laparoscopic (95.2%, *n* = 335). Extended resection was performed in 21.9% (*n* = 263) of patients, of which 14.3% (*n* = 172) underwent a multivisceral resection and 10.1% (*n* = 121) a vascular resection. Major complications occurred in 19.3% (*n* = 231) of patients. The median number of resected lymph nodes was 17 (IQR 10–26), and the median number of positive lymph nodes was 1 (IQR 1–3). The mean lymph node ratio was 0.12 (SD ± 0.17). Lymphovascular and perineural invasion were present in 55.7% (*n* = 668) and 72.4% (*n* = 869) of patients, respectively. Radical resection (R0) was obtained in 56.8% (*n* = 681) of patients. Adjuvant chemotherapy was administered in 59.5% (*n* = 714) of patients. Patient characteristics and outcomes are presented in Table 1.

**Table 1:** Patient characteristics and outcome

	Total cohort
	*N* = 1200
Age, years, median (IQR)	68 (61–75)
Female, *n* (%)	604 (50.3)
ASA score, *n* (%)	
ASA score I–II	789 (65.8)
ASA score III–IV	329 (27.4)
Unknown	82 (6.8)
Neoadjuvant treatment, *n* (%)	128 (10.7)
Neoadjuvant chemotherapy, *n* (%)	124 (10.3)
Neoadjuvant radiotherapy, *n* (%)	28 (2.3)
Resection of Gerota’s fascia, *n* (%)	360 (30.0)
Extended resection, *n* (%)	263 (21.9)
Multivisceral resection, *n* (%)	172 (14.3)
Vascular resection, *n* (%)	121 (10.1)
Appleby procedure, *n* (%)	13 (1.1%)
Blood loss, ml, median (IQR)	280 (100–550)
Operative time, minutes, median (IQR)	237 (180–295)
Clavien–Dindo grade III or higher, *n* (%)	231 (19.3)
Tumor size, mm, median (IQR)	32.0 (23–45)
Number of lymph nodes resected, median (IQR)	17.0 (10–26)
Number of lymph nodes positive, median (IQR)	1 (0–3)
Lymph node ratio, mean ± SD	0.12 ± 0.17
Lymphovascular invasion, *n* (%)	668 (55.7)
Perineural invasion, *n* (%)	869 (72.4)
Margin status, *n* (%)	
R0 (≥ 1 mm)	681 (56.8)
R1 (< 1 mm)	481 (40.1)
R2 (macroscopically not radical)	10 (0.8)
Unknown	28 (2.3)
AJCC stage^a^, *n* (%)	
I–II	1116 (93.0)
III	47 (3.9)
Adjuvant chemotherapy, *n* (%)	714 (59.5)

^a^According to the seventh AJCC definition

### Gerota’s Fascia Resection

Gerota’s fascia resection was performed in 30% (*n* = 360) of patients. Six of 34 (18%) centers resected Gerota’s fascia routinely. Patients undergoing Gerota’s fascia resection had larger median tumor size (35 mm, IQR 25–50 mm versus 32 mm, IQR 22–45 mm, *p* = 0.017), more extended resections (28% vs. 22%, *p* = 0.027), a higher lymph node yield (median 19, IQR 13–27 versus 17, IQR 9–27 nodes, *p* = 0.007), but a lower rate of R0 resection at circumferential margins (34% vs. 60%, *p* < 0.001). Tumors in the group with Gerota’s fascia resection had lower rates of lymphovascular invasion (59% vs. 67%, *p* = 0.010). There were no differences in major complications (19% vs. 16%, *p* = 0.191) between patients with and without Gerota’s fascia resection. The results of these analyses are presented in Table 2.

**Table 2:** Variables and outcomes stratified for Gerota’s fascia resection

Variable	Resection of Gerota’s fascia (*n* = 360)	No resection of Gerota’s fascia (*n* = 596)	*p* value
Involvement in other organs^a^, *n* (%)	50 (13.9)	61 (10.2)	0.089
Vascular involvement^b^, *n* (%)	103 (28.6)	163 (27.3)	0.084
Neoadjuvant treatment, *n* (%)	48 (13.3)	56 (9.4)	0.063
Neoadjuvant chemotherapy, *n* (%)	46 (12.8)	55 (9.2)	0.090
Neoadjuvant radiotherapy, *n* (%)	12 (3.3)	9 (1.5)	0.063
Minimally invasive approach, *n* (%)	76 (21.1)	178 (29.9)	0.003
Extended resection, *n* (%)	100 (27.8)	128 (21.5)	0.027
Tumor size, median, mm (IQR)	35 (25–50)	32 (22–45)	0.017
Overall R0 resection rate^c^	170 (47.2)	361 (60.6)	< 0.001
R0 resection rate at transection margin	269 (93.1)	446 (91.7)	0.509
R0 resection rate at circumferential margin	79 (21.9)	272 (45.6)	< 0.001
Lymph node yield, median, *n* (IQR)	19 (9–27)	17 (13–27)	0.007
Lymph node ratio, mean ± SD	0.12 ± 0.18	0.12 ± 0.16	0.700
Lymphovascular invasion, *n* (%)	200 (58.5)	364 (67.0)	0.010
Perineural invasion, *n* (%)	272 (79.5)	452 (81.3)	0.516
Major complications, *n* (%)	70 (16.1)	96 (19.4)	0.191

*IQR* interquartile range

^a^Defined as any additional organ involvement beyond the spleen

^b^Defined as any vascular involvement beyond the splenic vessels

^c^Defined as microscopic radical resection with a distance between the tumor and the margin of ≥ 1 mm

### Survival Analysis

The median follow-up period was 15 months (IQR 5–31 months), and 76 patients were lost to follow-up. In total, 484 patients died, and 640 patients were censored for survival analysis. The median overall survival was 30 months (95% CI, 27–33 months). Cumulative survival rates at 1-, 3-, and 5-year postoperatively were 80%, 42%, and 27% respectively.

**Fig. 1:**
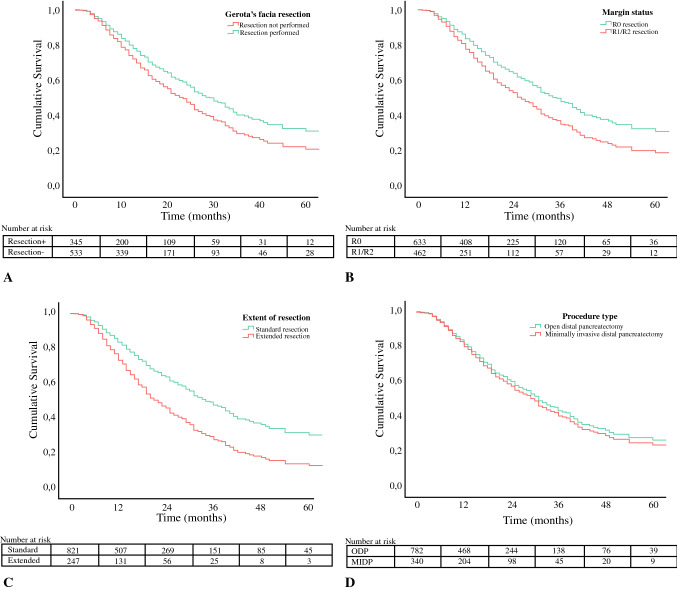
Adjusted Kaplan–Meier figures for overall survival stratified by surgical factor: **a** Gerota’s fascia resection, **b** margin status, **c** extent of resection, and **d** procedure type

None of the variables included in the Cox proportional hazard models violated the assumption of proportional hazards. At univariable analysis, Gerota’s fascia resection, extended resection, R0 resection, and lymph node ratio were associated with survival. At multivariable model analysis, Gerota’s fascia resection (HR 0.74; 95% CI 0.57–0.95; *p* = 0.019), R0 resection (HR 0.70; 95% CI 0.54–0.90; *p* = 0.006), and decreased lymph node ratio (HR 0.28; 95% CI 0.16–0.45; *p* < 0.001) were associated with improved overall survival, whereas extended resections (HR 1.74; 95% CI 1.33–2.32; *p* < 0.001) was associated with worse overall survival. Minimally invasive distal pancreatectomy did not improve survival as compared with open distal pancreatectomy (HR 1.14; 95% CI 0.87–1.49; *p* = 0.350). The results of the univariable and multivariable analyses are presented in Table 3. Kaplan–Meier curves stratifying for these surgical factors are shown in Fig. 1.

**Table 3:** Cox proportional hazard analyses for overall survival

Variable	Univariable analysis			Multivariable analysis		
	HR	95% CI	*p* value	HR	95% CI	*p* value
Resection of Gerota’s fascia	0.81	0.66–1.01	0.057	0.74	0.57–0.95	0.019
Minimally invasive DP	0.98	0.80–1.19	0.836	1.14	0.87–1.49	0.350
Extended resection^a^	1.68	1.38–2.05	**< **0.001	1.75	1.33–2.32	< 0.001
Tumor size > 2 cm	2.53	1.92–3.35	**< **0.001	1.87	1.28–2.74	0.001
R0 resection^b^ (R1 as reference)	0.68	0.57–0.82	**< **0.001	0.70	0.54–0.90	0.006
Lymph node ratio (decreasing)	0.27	0.18–0.39	**< **0.001	0.28	0.16–0.45	< 0.001
Lymphovascular invasion	1.51	1.24–1.84	**< **0.001	0.94	0.70–1.27	0.689
Perineural invasion	1.86	1.43–2.41	**< **0.001	1.71	1.16–2.53	0.007
AJCC stage III (I–II as reference)	2.38	1.62–3.50	**< **0.001	0.97	0.53–1.75	0.912
Adjuvant chemotherapy	0.66	0.54–0.81	**< **0.001	0.67	0.52–0.87	0.003

*HR* hazard ratio, *CI* confidence interval, *ASA* American Society of Anesthesiologists, *DP* distal pancreatectomy, *AJCC* American Joint Committee against Cancer

^a^Defined as additional organ resection beyond the spleen and as any vascular resection beyond the splenic vessels

^b^Defined as microscopic radical resection with a distance between the tumor and the margin of ≥ 1 mm

Gerota’s fascia resection remained independently associated with overall survival in the sensitivity analysis, excluding the 25% largest tumors (HR 0.68; 95% CI 0.49–0.94; *p* = 0.019). The results are presented in Supplementary Table 1.

## Discussion

This international multicenter cohort study including 1200 patients who underwent distal pancreatectomy for PDAC in 34 centers from 11 European countries plus the USA identified Gerota’s fascia resection, R0 resection, and decreased lymph node ratio as surgical factors associated with improved overall survival. Extended resection was independently associated with worse overall survival. Minimally invasive distal pancreatectomy did not improve overall survival.

Currently, resection of Gerota’s fascia is not standard surgical practice during distal pancreatectomy. This is shown by the present study as surgeons only included this step in 30% of the procedures. Accordingly, 70% of 237 pancreatic surgeons in a recent European survey considered Gerota’s fascia resection not an essential step during an oncological radical distal pancreatectomy for PDAC.^21^ According to the ISGPS guidelines for extended pancreatic resection, Gerota’s fascia resection could be considered during distal pancreatectomy, but is not advised as a standard approach.^16^

The aim of Gerota’s fascia resection is to achieve higher rates of R0 resection at the posterior margin and, simultaneously, achieve a better lymph node dissection as described by Strasberg and, for minimally invasive distal pancreatectomy, by Abu Hilal.^11^,^12^ The present study confirmed the higher lymph node yield but found a surprising lower rate of R0 resection at the circumferential margin with Gerota’s fascia resection (Table 2). These lower rates of R0 resection may actually be related to the higher tumor-positive anterior pancreatic margin rate rather than the actual circumferential surgical posterior margin, as slightly larger tumors and more extended resections were found in the Gerota’s fascia resection group. Despite these differences, Gerota’s fascia resection was independently associated with improved overall survival, which remained when excluding the 25% largest tumors. These findings underline the potential relevance of Gerota’s fascia resection, which the authors suggest to be integrated in standard practice when performing a distal pancreatectomy for PDAC.

The assessment of resected lymph nodes remains crucial for disease staging, and therefore adjuvant treatment options, in this group of patients. The number of retrieved lymph nodes has been associated with improved survival outcomes, whereas the number of positive lymph nodes and increasing lymph node ratio have been associated with worse survival in previous reports.^8^,^9^,^22^^–^27 In accordance, the present study found a decreased lymph node ratio to be associated with improved overall survival. To achieve accurate N staging and low lymph node ratios, it is important to resect at least a minimum number of 11 lymph nodes, as agreed on by worldwide experts during the set-up of the DIPLOMA trial.^28^ For pancreatic tail tumors, stations 10, 11, and 18 should be resected, whereas an additional resection of stations 8 and 9 should be performed for pancreatic body tumors. It is however uncertain whether resecting more lymph nodes is a therapeutic measure to improve overall survival. Several randomized trials in patients undergoing pancreatoduodenectomy for PDAC found that more extensive lymphadenectomy does not improve overall survival.^29^ A lower lymph node ratio may therefore also reflect less invasive disease biology.

Although the first randomized controlled trials (RCTs) on minimally invasive versus open distal pancreatectomy (LEOPARD and LAPOP trials) demonstrated benefits of minimally invasive over open distal pancreatectomy,^30^,^31^ the use of minimally invasive distal pancreatectomy for pancreatic cancer is still being questioned. In a European survey, 31% of respondents expected inferior oncological outcomes after minimally invasive distal pancreatectomy as compared with open distal pancreatectomy for PDAC.^21^ Several comparative studies, however, reported comparable median survival estimates between both approaches ranging between 24 and 32 months.^3^,^4^,^6^,^7^,^9^,^32^^–^34 Accordingly, the present study demonstrated a median overall survival of 30 months in open distal pancreatectomy and 31 months in minimally invasive distal pancreatectomy. Furthermore, based on the findings in the present study and other studies, the minimally invasive approach does allow for Gerota’s fascia resection, R0 resection, and adequate lymph node yield.^11^,^15^,^35^^–^37 Although these results seem promising, more data from randomized controlled trials should be awaited. Currently, three randomized controlled trials are ongoing to compare minimally invasive and open distal pancreatectomy in patients with PDAC and should provide more insight in this matter: the DIPLOMA trial, performed in Europe and the USA (ISRCTN44897265), one trial in China (NCT03792932), and one trial in Korea (NCT03957135). The DIPLOMA trial includes Gerota’s fascia as routine step during resection in both minimally invasive and open distal pancreatectomy.

This study has several limitations which should be taken into account. First, due to the retrospective multicenter design and long inclusion period of this study, heterogeneity will be present regarding surgical approach, histopathology procedures, and the use of chemotherapy, which could influence outcomes. A major limitation to the retrospective design is the assessment of Gerota’s fascia resection, since this was either reported specifically or deduced from other data in the surgical report, which might have led to underreporting and bias. Selection bias, however, would be expected to worsen survival with Gerota’s fascia resection since this would typically be done in case of larger tumors, but mixed oncological characteristics were found in this group of patients. Future research should focus on these findings. Second, data regarding tumor grade are missing in this study, which has been suggested as a predictor for overall survival.^3^,^9^ Third, the relatively short follow-up may have led to less accurate survival predictions on the Kaplan–Meier estimates and Cox proportional hazard analyses. Strengths of this study include the large cohort from a large number of countries spanning both Europe and the USA with a focus on surgical aspects of distal pancreatectomy for PDAC.

In conclusion, this international cohort identified Gerota’s fascia resection, R0 resection, and decreased lymph node ratio as factors associated with improved overall survival. During DP for PDAC, surgeons should strive for R0 resection and adequate lymphadenectomy but also consider Gerota’s fascia resection in their routine surgical approach.

### Electronic supplementary material

Below is the link to the electronic supplementary material.

Supplementary material 1 (DOCX 14 kb)

## Appendix

Variables collected in this study

Baseline characteristics

- Name of hospital
- Age
- Sex
- Body mass index (kg/m^2^)
- Prior abdominal surgery
- Chronic pancreatitis
- American Society of Anesthesiologists score
- Preoperative imaging:
  - Tumor size
  - Tumor location
  - Involvement of other organs
  - Vascular involvement
  - Metastasis
- Neoadjuvant treatment:
  - Radiotherapy and/or chemotherapy

Surgical details

- Procedure type (laparoscopic, robotic, or open)
- Conversion
- Gerota’s fascia resection
- Splenectomy
- Extended resection: multivisceral and/or vascular resection
- Operative time
- Blood loss
- Blood transfusion

Histopathology

- Final diagnosis
- Tumor size
- Distance of tumor to margin (in mm), both transection and circumferential
- Lymph nodes resection
- Lymph node status
- Lymphovascular and/or perineural invasion
- Tumor stage: TNM classification (7^th^ definition)

Postoperative data

- Clavien–Dindo classification
- Postoperative pancreatic fistula (POPF)
- Delayed gastric emptying (DGE)
- Postpancreatectomy hemorrhage (PPH)
- Surgical site infection
- Reintervention
- Intensive care admission
- Length of stay
- Readmission

Follow-up data

- Adjuvant chemotherapy
- Recurrence
- Mortality
- Length of follow-up
- Date of death
