# Supplementary material for: Predictors for Survival in an International Cohort of Patients Undergoing Distal Pancreatectomy for Pancreatic Ductal Adenocarcinoma
Source: Ann Surg Oncol. 2020 Jun 25;28(2):1079–87. doi: 10.1245/s10434-020-08658-5 (PMC7801299; doi:10.1245/s10434-020-08658-5)
Supplement: Supplementary file 1 — Supplementary material 1 (DOCX 14 kb) [file 10434_2020_8658_MOESM1_ESM.docx]

| **SUPPLEMENTARY TABLE 1** *Cox proportional hazard analyses after excluding the 25% largest tumors* | | | | | | | |
| --- | --- | --- | --- | --- | --- | --- | --- |
|  | **Univariable analysis** | | |  | **Multivariable analysis** | | |
| **Variable** | **HR** | **95% CI** | ***p*-Value** |  | **HR** | **95% CI** | ***p*-Value** |
| Resection of Gerota’s fascia | 0.77 | 0.60–1.01 | 0.055 |  | 0.68 | 0.49–0.94 | **0.019** |
| Minimally invasive DP | 1.00 | 0.79–1.27 | 0.986 |  | 1.15 | 0.83–1.61 | 0.399 |
| Extended resection^†^ | 1.57 | 1.20–2.05 | 0.001 |  | 1.75 | 1.19–2.58 | **0.005** |
| Tumor size > 2 cm | 2.28 | 1.71–3.03 | < 0.001 |  | 1.65 | 1.10–2.46 | **0.015** |
| R0 resection^‡^ (R1 as reference) | 0.75 | 0.60–0.94 | 0.013 |  | 0.72 | 0.52–0.99 | **0.045** |
| Lymph node ratio (decreasing) | 0.12 | 0.06–0.22 | 0.001 |  | 0.08 | 0.03–0.21 | **< 0.001** |
| Lymphovascular invasion | 1.45 | 1.14–1.84 | 0.003 |  | 0.83 | 0.56–1.22 | 0.336 |
| Perineural invasion | 1.75 | 1.29–2.39 | < 0.001 |  | 1.87 | 1.16–3.00 | **0.010** |
| AJCC stage III (I–II as reference) | 2.59 | 1.59–4.24 | < 0.001 |  | 0.69 | 0.31–1.52 | 0.354 |
| Adjuvant chemotherapy | 0.74 | 0.57–0.98 | 0.033 |  | 0.71 | 0.51–0.98 | 0.036 |
| *HR* hazard ratio, *CI* confidence interval, *ASA* American Society of Anaesthesiologists, *DP* distal pancreatectomy, *AJCC* American Joint Committee against Cancer  ^†^Defined as additional organ resection beyond the spleen and as any vascular resection beyond the splenic vessels  ^‡^Defined as microscopic radical resection with a distance between the tumor and the margin of ≥1 mm | | | | | | | |
